# Self-reported health status and disease-specific quality of life one year after treatment for peripheral arterial disease in clinical practice

**DOI:** 10.1186/s12955-020-01477-y

**Published:** 2020-07-17

**Authors:** Anne Sofie F. Larsen, Anne Therese Reiersen, Inger Helene Nådland, Jarlis Wesche

**Affiliations:** 1 Department of Radiology, Ostfold Hospital Trust, PB300, 1714, Grålum, Norway; 2 Department of vascular surgery, Ostfold Hospital Trust, Grålum, Norway; 3 Department of Vascular and Thoracic Surgery, Akershus University Hospital, Lørenskog, Norway; 4 Institute of Clinical Medicine, Faculty of Medicine, University of Oslo, Oslo, Norway

**Keywords:** Peripheral arterial disease, Quality of life, Patient reported outcome measures, Intermittent claudication, Endovascular procedures, Vascular surgical procedures

## Abstract

**Background:**

VascuQoL-6 (VQ-6) is a disease-specific quality of life (QoL) instrument validated for use in clinical practice and vascular registries before and after treatment for peripheral arterial disease (PAD). To improve future interpretation of self-reported outcome, an unselected cohort was followed through one year to provide observational data after both conservative and invasive treatment.

**Methods:**

Consecutive patients with intermittent claudication (IC) or critical limb ischemia (CLI) were included. All patients completed VQ-6 and Short Form-36 (SF-36), and were evaluated with ankle-brachial index (ABI) measurement pre- and post-exercise, a constant load treadmill test and clinical consultation at baseline and after one year. Change statistics and correlation analysis were used to describe self-reported outcome after conservative and invasive treatment for PAD.

**Results:**

One hundred seventy-one patients with peripheral arterial disease (PAD) were included, 70 (41%) female. 147 (86%) of the patients suffered from IC. 136 (80%) patients had one-year follow up, death, amputation and withdrawal were the major causes of loss to follow-up. Forty-eight patients (35%) evaluated their health to be unchanged compared to one year ago. There was a strong correlation between self-reported general health status based on SF-36 item 2 and VQ-6 summary score (Spearmans rho = − 0.536). Patients admitted to invasive intervention (endovascular or surgery) improved in all domains of SF-36, and in the physical component summary score (SF-36 PCS). Patients admitted to best medical treatment, smoking cessation and walking exercise (conservative group) improved only in the physical domains. There was significant improvement in VQ-6 summary score for both groups, mean 2.20 (95%CI 1.14–3.27) in the conservative group, 4.68 (95%CI 3.67–5.70) in the invasive group. VQ-6 sum score improved more than four points for 56% in the invasive group, 36% in the conservative group.

**Conclusions:**

Treatment for symptomatic PAD, both invasive and conservative, improves self-reported health status and disease specific QoL after one year. Interpretation of patient-reported outcome measured with VQ-6 after surgery or endovascular treatment must be seen in light of the improvement from conservative treatment alone.

**Trial registration:**

ISRCTN14846962 (retrospectively registered).

## Background

Patient reported outcome measures (PROMS), such as self-reported health status and disease-specific quality of life (QoL), are regarded as important and valuable outcome variables after treatment for peripheral arterial disease (PAD), supplementing the clinical evaluation and evaluation of peripheral arterial pressure measurements and walking tests [1]. For patients with intermittent claudication, self-reported outcome measures can capture improvement beyond clinical testing.

VascuQoL-6 (VQ-6) is a short version of the disease-specific quality of life instrument VascuQoL-25 [2, 3], intended both for intermittent claudication (IC) and critical limb ischemia (CLI). VascuQoL-6 was found to be valid, reliable and responsive to change [4]. The VascuQoL-6 study aimed both to validate the VQ-6-instrument based on registrations at baseline and after 4 weeks, and to investigate the development in self-reported health status, measured with SF-36, and disease-specific QoL, measured with VQ-6, after one year for consecutive patients treated for peripheral arterial disease in clinical practice.

Self-reported health status (SF-36) of PAD patients are substantially reduced at baseline compared to healthy people in the same age group, but show improvement after both invasive (endovascular/surgery) and conservative treatment [4, 5]. In order to improve interpretation of future self-reported outcome measured with VQ-6 from procedural registries in vascular surgery, the validation study cohort was followed through a year as an observational study. There is a lack of knowledge of how QoL measured with VQ-6 change for patients who receive conservative treatment with best medical treatment (BMT), smoking cessation advice and walking exercise.

## Purpose

The aim of this study was to investigate change in self-reported health status measured with SF-36 and VQ-6 one year after treatment for PAD, in an unselected population of conservatively and invasively treated patients.

## Methods

### Inclusion and exclusion

**Fig. 1:**
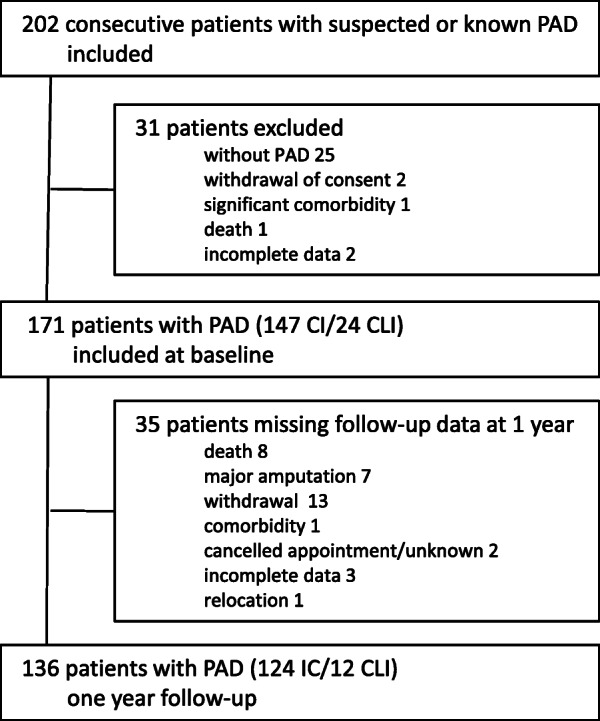
Inclusion and exclusion of patients in the VascuQoL-6 study. PAD –Peripheral arterial disease, IC-intermittent claudication, CLI –critical limb ischemia

Consecutive patients with new referral for evaluation of peripheral arterial disease (IC or CLI) at the vascular surgery department at two different hospitals were invited to participate in the VascuQol-6 study, based on the information given by the referring physician. The inclusion period ran from August 2014 to August 2015. Inclusion, exclusion and follow-up of patients are described in detail in a previously published paper [4], and summarized in Fig. 1.

Patients received written information about the study and the two questionnaires, VQ-6 and SF-36, by mail before the scheduled appointment at the outpatient clinic. The questionnaires were not available to the treating physician.

### Work up

In the vascular laboratory, arterial pressures were measured with a hand held Dopplerdevice, and the ankle-brachial index (ABI) was calculated. Patients with claudication were tested on a constant load treadmill with a speed of 2.5 km/h and no inclination. A post-exercise ABI drop exceeding 0.1 was regarded significant. Intermittent claudication distance (ICD) and maximum walking distance (MWD) were registered in meters. There is a ceiling effect at 416 m, as the test was terminated after 10 min for all patients.

Risk factors, comorbidity, medication and Fontaine classification [6] were registered by the vascular surgeon during the clinical consultation.

### Follow-up

All eligible patients from the VascuQoL-6 study were scheduled for a new consultation with completion of questionnaires, arterial pressure measurements, treadmill-test and clinical evaluation after one year. The conservative treatment group included patients with best medical treatment (BMT) alone (information about the disease, the value of walking exercise and medical treatment) and patients with additional referral for supervised exercise therapy (SET). The invasive treatment group included patients with endovascular and/or vascular surgical procedures in addition to BMT. The referral algorithm for imaging and invasive treatment was unaltered from usual practice.

### VascuQoL-6

VascuQoL-6 (VQ-6) is a short disease-specific QoL instrument intended for use in clinical practice and vascular registries [3, 4, 7]. Each of the six items scores from one to four, sum score range is from six to 24, and a higher score indicates better health.

### SF-36

**Fig. 2:**
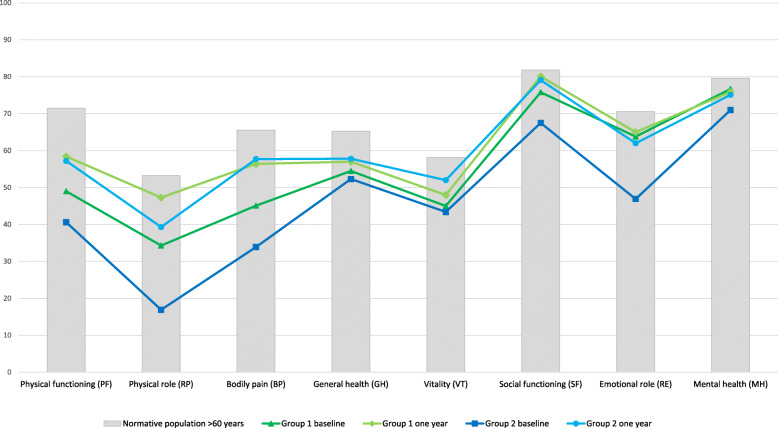
Self-reported health status at baseline and one year after conservative (Group 1) or invasive (Group 2) treatment for peripheral arterial disease. Mean value in the domains of Short Form-36. For comparison Norwegian norm population aged 60 years and over (Loge 1998)

SF-36 (version 1) was used to measure general self-reported health status. This instrument has been used in prior validation of VascuQoL-25 [8–11] and in a range of PAD studies [12–15] . Subscale (PF –physical functioning, RP –physical role, BP –bodily pain, GH –general health, VT –vitality, SF -social functioning, RE –emotional role, MH –mental health) and component summary scoring (PCS –physical component score, MCS –mental component score) was performed using Qualimetric Health Outcomes Scoring Software 4.0, using the original scoring method [16]. This software uses the 1998 US norm population for calculation of component summary scores, as US norm has been recommended for western countries [17]. Data from the Norwegian norm population from 1998 [18] was used in Fig. 2 for illustrative purposes.

The subscale scores of SF-36 range from 0 to 100, and the highest score indicates no health-related reduction of QoL. The component summary scores relates to the normative population (mean 50, SD 10), and a score lower than 50 indicates lower QoL than the normative population.

### Statistic methods

All statistics were calculated using Statistical Package for the Social Sciences version 21 (IBM/SPSS Inc., Armonk, NY, USA). Change analysis was done using t-test for normally distributed continuous data, Wilcoxon signed rank test for non-parametric continuous data and McNemar’s test for dichotomous data. The relationship between perceived general health change and change in VQ-6 sum score was investigated using non parametric statistics, Spearmans rank correlation coefficient, interpreted using Cohens criteria (1988, p79–81) (small: rho 0.1–0.29, medium 0.3–0.49, large > 0.5).

#### Missing data

Analysis was performed using all available data at baseline and during follow-up. For missing items for VQ-6 (2 patients, 1 and 2 missing items, conservative treatment group) we did a single imputation using the last value carried forward. One patient missed three items after treatment, and the sum score of VQ-6 was omitted from analysis. For SF-36, imputation was done using the scoring software (Qualimetric Health Outcomes Scoring Software 4.0). The mean subscale score is used if the patient has answered more than half of the items in the domain.

## Results

One hundred seventy-one patients were included, 41% female. Some 53 (31%) had a previous history of evaluation or treatment for PAD. Patient characteristics are given in Table 1. Of the patients, 147 (86%) were claudicants (Fontaine IIA/B) and 24 (14%) suffered from critical limb ischemia (Fontaine III and IV). A total of 142 (83%) participated in a treadmill test. At baseline, 116 (68%) received treatment with platelet inhibitors and statins.

**Table 1:** Patients characteristic (*n* = 171)

	N (eligible for analysis)	Percent	Median (range)
Age	171		
Male		59.1%	70 (47–89)
Female		40.9%	71 (44–89)
BMI	162		26.6 (16.4–41.2)
Smoking^a^	169	60.9%	
Diabetes	171	21.1%	
Impaired renal function	137		
eGFR< 60		21.2%	
eGFR< 45		8.8%	
Anti-hypertensive treatment	151	74.8%	
Cerebrovascular disease	171	15.8%	
Cardiovascular disease	171	39.2%	
Chronic obstructive pulmonary disease	171	18.1%	
Other comorbidity	171	14.6%	
Work status	168		
Paid work		15.5%	
Sick leave or disability pension		18.4%	
Retired or unpaid work		66.1%	

^a^Smoking or previous smoking within 5 years

One hundred thirty-six patients had follow up after one year, 12 (9%) of these were treated for CLI, the remaining IC. Half of the 24 CLI patients included at baseline were lost to follow up (major amputation 6, death 3, and other 3). Arterial pressures, treadmill walking capacity and QoL-summary scores at baseline and one year follow up is shown in Table 2.

**Table 2:** Quality of life summary scores, arterial pressure indices and walking capacity at baseline and one year follow-up

	All participants (*n* = 171)	Conservative treatment (*n* = 59)			Invasive treatment (*n* = 77)			No follow-up (*n* = 35)
	Baseline	Baseline	Follow-up		Baseline	Follow-up		Baseline
	**Mean (95% CI)**	**Mean (95% CI)**	**Mean (95% CI)**	***p***	**Mean (95% CI)**	**Mean (95% CI)**	***p***	**Mean (95%CI)**
**VQ-6 sum**	12.7 (12.2–13.3)	14.4 (13.5–15.2)	16.6 (15.5–17.6)	*0.001*^*b*^	12.1 (11.4–12.7)	16.8 (15.7–17.8)	*0.001*^*b*^	11.4 (10.1–12.7)
**SF-36 PCS**	33 (32–34)	35.2 (33.1–37.3)	40.1 (37.2–42.9)	*0.001*^*b*^	32.0 (30.4–33.5)	39.7 (37.1–42.3)	*0.001*^*b*^	31.6 (29.5–33.7)
**SF-36 MCS**	48 (47–50)	51.5 (48.4–54.6)	50.3 (47.6–53.0)	*0.385*^*b*^	48.4 (45.8–51.0)	50.4 (47.9–52.8)	*0.141*^*b*^	43.5 (39.2–47.8)
**ABI**^**a**^	0.62 (0.59–0.65)	0.67 (0.63–0.71)	0.66 (0.61–0.71)	*0.781*^*b*^	0.58 (0.53–0.63)	0.76 (0.71–0.82)	*0.001*^*b*^	0.60 (0.53–0.67)
**ABI pe**^**a**^	0.47 (0.43–0.51)	0.56 (0.5–0.61)	0.60 (0.53–0.67)	*0.110*^*b*^	0.39 (0.33–0.45)	0.70 (0.61–0.78)	*0.001*^*b*^	0.44 (0.31–0.56)
	**Median (IQ)**	**Median (IQ)**	**Median (IQ)**		**Median (IQ)**	**Median (IQ)**		**Median (IQ)**
**ICD**	87 (46–133)	110 (62–164)	160 (100–230)	*0.022*^*c*^	61 (36–100)	90 (50–130)	*0.011*^*c*^	95 (50–160)
**MWD**	400 (164–410)	410 (300–420)	408 (400–410)	*0.567*^*c*^	240 (110–410)	406 (344–410)	*0.001*^*c*^	287 (190–410)

*VQ-6* Vascular Quality of Life Questionnarie-6, *SF-36* Short Form-36, *PCS* Physical component summary score, *MCS* Mental component summary score, *ABI* ankle-brachial index, *pe* postexercise, *ICD* intermittent claudication distance, *MWD* maximum walking distance, *IQ* interquartile range (25th–75th percentile)

^a^symptomatic leg

^b^two-tailed t-test for normally distributed data, *p* < 0.05 was regarded significant

^c^Wilcoxon signed rank test for non-normally distributed data, *p* < 0.05 was regarded significant

### Self-reported health status

Based on item 2 in SF-36 (“Compared to one year ago, how would you rate your health in general now?”), 30 patients (22%) reported their health to be much improved, 29 (21%) somewhat improved, 48 (35%) unchanged, 25 (18%) somewhat worse, and 6 (4%) much worse.

There was a strong correlation between item 2 in SF-36 and change in VascuQoL-6 sum score, Spearmans rho = − 0.536, n = 136, *p* < 0.01, where a large improvement in sum score was associated with better health now than one year ago.

For the patients who reported an unchanged health status the mean change in VQ-6 summary score was 2.3 (95%CI: 1.3–3.25). For the 56 (41%) patients where the vascular surgeon evaluated the status to be unchanged, the mean change in VQ-6 summary score was 2.4 (95%CI: 1.44–3.45).

### Conservative treatment group

**Fig. 3:**
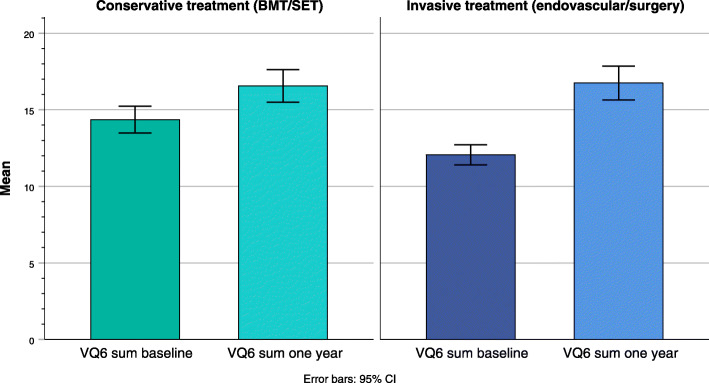
Disease specific quality of life measured with VascuQol-6 at baseline and one year after treatment for peripheral arterial disease. Mean summary score with 95% CI

**Fig. 4:**
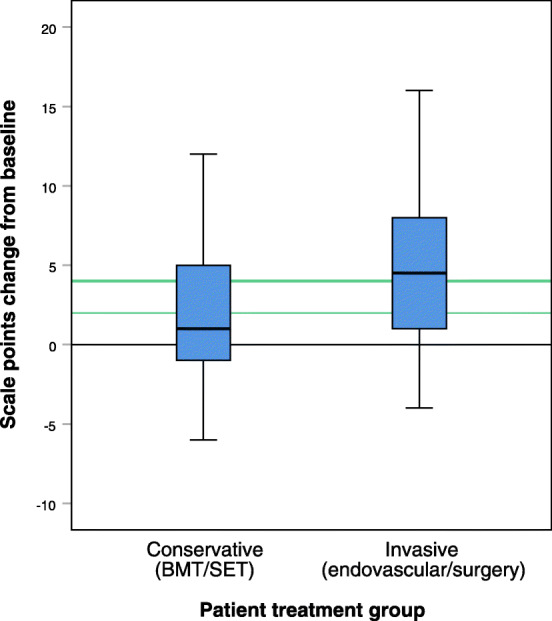
VasuQol-6 summary score change one year after treatment for peripheral arterial disease. Boxplot with median and quartiles. Green lines mark two and four scale points improvement

Fifty-nine patients were treated with a conservative approach (IC/CLI: 58/1). There was significant improvement in self-reported physical health status and disease-specific QoL, as shown in Table 2.The mean change in SF-36 subscale score was largest for the physical domains (PF-physical functioning, RP-Physical role and BP –Bodily pain), as shown in Fig. 2. Figure 3 show mean score of VQ-6 at baseline and after treatment. Change in VQ-6 summary score ranged from − 6 to 12. Nine patients (15%) had reduced summary score by 2 or more scale points, 23 patients (39%) had unchanged score (− 1 to 1), and 27 patients (46%) had increased score by 2 or more scale points. Of these, 21 (36%) had increased their summary score by 4 points or more, as shown in Fig. 4.

There was a high proportion of patients able to complete the 10 min walking test at baseline, 38 (64%), increasing to 43 (73%) after one year. There was no significant improvement in ABI at rest or after exercise. Of the 59 patients, 14 completed 18 weeks of supervised exercise treatment with a physical therapist. There were no significant differences between patients with SET compared to BMT alone at baseline or follow up**.**

### Invasive treatment group

Among the 136 patients with QoL follow-up data after one year, 77 patients had been treated with endovascular or surgical procedures (IC/CLI: 66/11) in addition to BMT. Ten patients received both endovascular treatment (PTA/stent) and surgical treatment (by-pass/thrombendarterectomy), 54 patients endovascular treatment and 13 patients surgical treatment. In the group with CLI seven patients received endovascular treatment and four surgical treatments.

There was significant improvement in self-reported physical health status and disease-specific QoL, as shown in Table 2. The mean change in SF-36 subscale score was largest for the physical domains (PF-physical functioning, RP-Physical role and BP –Bodily pain), but also significant for the mental domains (VT-Vitality, SF –Social functioning and RE –Emotional role), as shown in Fig. 2. Figure 3 show mean score of VQ-6 at baseline and after treatment. Change in VQ-6 summary score ranged from − 4 to 16. Four patients (5%) had reduced summary score by 2 or more scale points, 16 patients (21%) had unchanged score (− 1 to 1). Some 56 patients (73%) had increased score by 2 or more scale points, and 43 (56%) had increased their summary score by 4 points or more, as shown in Fig. 4. In addition, there was significant improvement in ABI at rest and after exercise, and in walking capacity. At baseline 24 patients (31%) completed the 10 min walking test, increasing to 46 (60%) after one year.

## Discussion

VascuQoL-6 was developed especially for clinical use and for use in vascular registries, where self-reported end points are increasingly important to evaluate outcome. Traditionally, quality of care was monitored in vascular registries by surveillance of adverse events and patency of the vascular reconstruction, but did not include whether life was improved for the patient. A validated instrument used to measure patient-reported outcome allows aggregation of data on a group level, and can be used to picture the treatment’s success in lessening symptoms. In clinical use, in the individual patient, the instrument can be used to evaluate key symptoms and how the patient perceives the impact on daily life.

Improvement or deterioration as shown by VQ-6 must, nevertheless, be interpreted with care. This study shows that both treatment groups improve their disease specific QoL as well as their health status after one year. Expected improvement from conservative treatment must thus be taken into account when interpreting outcome after invasive treatment, as not all improvement can be attributed to the invasive procedure.

Since this study was conducted, the validity and test retest reliability of VQ-6 have been tested in a Swedish population of mainly CLI (70%) [19], and the validity in a Brazilian PAD population with intermittent claudication [20]. Experience with the instrument are emerging also from the national vascular registry of Sweden, Swedvasc [21]. VascoQoL-6 has recently been introduced in NORKAR, the national Norwegian vascular registry.

The two groups of conservatively treated and invasively patients in our study are not directly comparable, as there was a clinically driven indication for choice of treatment, and the invasively treated patients thus had a significantly lower level of function and more advanced disease. Many of the conservatively treated patients did not improve their walking distance due to this selection bias, as their baseline values were close to the tests maximum of 416 m. This selection bias probably also influences the results from SET versus conservative treatment alone, as this study fails to demonstrate the benefit of SET shown in other studies [22]. Compared to arterial pressures and ABI, VQ-6 can detect improvement also in the absence of revascularization. As expected, ABI did not improve in the conservative treatment group, as increased blood flow and reduced symptoms are a result of development of collaterals, with persistent stenosis or occlusion of the artery.

A population-based study from the Swedish national vascular registry, Swedvasc, has indicated 1.7–2.2 scale steps as a minimally important difference (MID), and 3.5–4.5 scale steps as a substantial clinical benefit [7]. MID was estimated using two different methods in the validation study [4]. One method (0.5 SD) gave a value of 1.725 scale points, the other (95%CI) 0.82 for improvement and − 1.38 for deterioration [23, 24]. To use the self-reported health status, SF-36 item 2, after one year to estimate MID will probably be wrong, as the recall bias will be too large. But, the improvement in VQ-6 summary score for the “unchanged” group (2.3) supports that the earlier proposal of two points of change as indicative and four points as a certain change [4].

Self-reported health status based on SF-36 will include comorbidity and reduced QoL from other conditions than the lower extremity arterial disease. Nevertheless, the correlation between SF-36 and VQ-6, indicates that for this patient population, the morbidity from the arterial insufficiency is the main cause of change in self-reported health status.

The qualitative analysis in the Swedish study, published after the validation study, show item four to be vulnerable for emotional status, and the term “concern” was evaluated to be rather weak, the authors suggest “anxious” and “worried” as stronger terms [19]. This can influence the interpretation of the results in our study, as confusion about one item may add or subtract as much as three points to the VQ-6 summary score. In item one, the patients report some confusion regarding the content of the term “activities” [19]. This term was broadened in the Norwegian translation to “activities and daily activities of living” after the cognitive evaluation by patients and health professionals [4].

The number of patients lost to follow up at one year reflects both the increased mortality and morbidity associated with this disease, also among patients with presumptive stable disease [1, 25], as well as the advanced age of the patients. Only half of the original included patients with the most advanced form of the disease, critical limb ischemia (CLI), completed the trial. This means that the results presented here probably are most relevant in interpreting outcome after treatment for IC. Further studies are warranted and future use of VQ-6 in randomized trials would improve the basis for clinical interpretation.

## Conclusions

Treatment for symptomatic PAD, both invasive and conservative, improves self-reported health status and disease specific QoL after one year. The natural history and development of the disease and the improvement from conservative treatment alone must be taken into account while interpreting patient-reported outcome in vascular registries.

## Data Availability

The datasets used and/or analyzed during the current study are available from the corresponding author on reasonable request.

## Abbreviations

ABI: ankle-brachial index
BMT: best medical treatment
CLI: critical limb ischemia
IC: intermittent claudication
ICD: intermittent claudication distance
MID: minimally important difference
MWD: maximum walking distance
PAD: peripheral arterial disease
PROMS: patient reports outcome measures
QoL: quality of life
SET: supervised exercise therapy
SF-36: Short Form-36, subscales (PF physical functioning, RP physical role, BP bodily pain, GH general health, VT vitality, SF social functioning, RE emotional role, MH mental health) and component summary scoring (PCS physical component score, MCS –mental component score)
VQ-6: VascuQoL-6 --Vascular Quality of Life questionnaire, short version
